# The floating knee: epidemiology, prognostic indicators & outcome following surgical management

**DOI:** 10.1186/1752-2897-1-2

**Published:** 2007-11-26

**Authors:** Ulfin Rethnam, Rajam S Yesupalan, Rajagopalan Nair

**Affiliations:** 1 Department of Orthopaedics, Glan Clwyd Hospital, Bodelwyddan, UK; 2 Department of Orthopaedics, Glan Clwyd Hospital, Bodelwyddan, UK; 3 Department of Orthopaedics, St John's medical college, Bangalore, India; 4 11 Ffordd Parc Castell, Bodelwyddan, Rhyl, LL18 5WD, UK

## Abstract

**Background:**

Floating Knee injuries are complex injuries. The type of fractures, soft tissue and associated injuries make this a challenging problem to manage. We present the outcome of these injuries after surgical management.

**Methods:**

29 patients with floating knee injuries were managed over a 3 year period. This was a prospective study were both fractures of the floating knee injury were surgically fixed using different modalities. The associated injuries were managed appropriately. Assessment of the end result was done by the Karlstrom criteria after bony union.

**Results:**

The mechanism of injury was road traffic accident in 27/29 patients. There were 38 associated injuries. 20/29 patients had intramedullary nailing for both fractures. The complications were knee stiffness, foot drop, delayed union of tibia and superficial infection. The bony union time ranged from 15 – 22.5 weeks for femur fractures and 17 – 28 weeks for the tibia. According to the Karlstrom criteria the end results were Excellent – 15, Good – 11, Acceptable – 1 and Poor – 3.

**Conclusion:**

The associated injuries and the type of fracture (open, intra-articular, comminution) are prognostic indicators in the Floating knee. Appropriate management of the associated injuries, intramedullary nailing of both the fractures and post operative rehabilitation are necessary for good final outcome.

## Background

The incidence of fractures resulting from motor vehicle accidents is on the rise. As a by-product of the horsepower race, high velocity accidents are now more common. Such accidents produce violent and complex injuries. Frequently, multiple fractures are produced in the same extremity, adding new dimensions to the problems of their management.

Floating Knee is the term applied to the flail knee joint segment resulting from a fracture of the shaft or adjacent metaphysis of the ipsilateral femur and tibia [1]. The fractures range from simple diaphyseal to complex articular types. This complex injury has increased in proportion to population growth, number of motor vehicles on the road, and high speed traffic. Although the exact incidence of the floating knee is not known, it is an uncommon injury. The largest series reported in the literature was of 222 patients over 11 years [2]. This injury is generally caused by high-energy trauma and the trauma to the soft tissues is often extensive. There also may be life-threatening injuries to the head, chest or abdomen and a high incidence of fat embolism. Management of this injury has been variously described in the literature [3-8].

The purpose of our study was to determine the outcome of patients after surgical management of the Floating Knee and identify prognostic factors for this injury.

## Methods

This was a prospective study conducted at a tertiary care trauma centre after approval of the Research and Ethics committee over a 3 year period (1998 – 2001). All floating knee injuries that were managed surgically during the study period were included. Children and floating knee injuries managed conservatively were excluded from the study.

**Figure 1:**
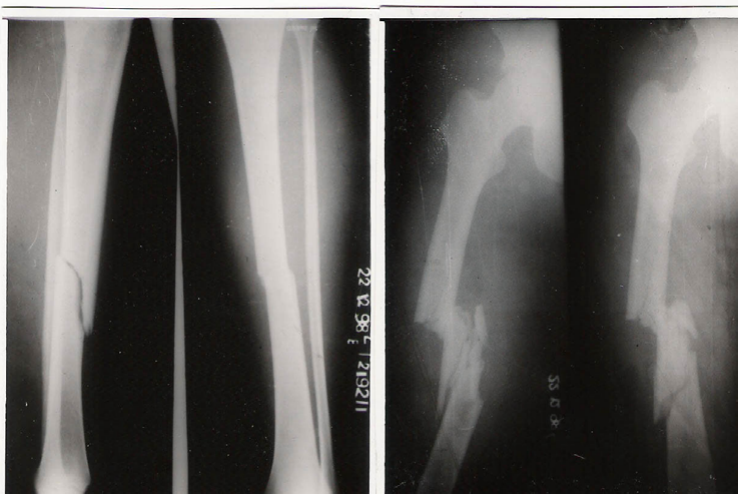
Type 1 Floating knee (Blake & McBryde classification).

Our study included 29 patients with 29 floating knee injuries, 27 males and 2 females [Table 1]. Initial management involved resuscitation and haemodynamic stabilisation of the patient, splinting of the affected limb in a Thomas splint and a thorough secondary survey to identify other injuries. Radiographs of the chest, pelvis, affected lower limb including all its joints and other suspected bony injuries were done. Open fractures were classified according to Gustilo & Anderson's classification [9]. Initial wound toilet, tetanus immunisation and antibiotic therapy was initiated for open fractures. The floating knee injury was classified according to Blake & McBryde's Classification [10] [Table 2] [Figure 1, 2 &3].

**Figure 2:**
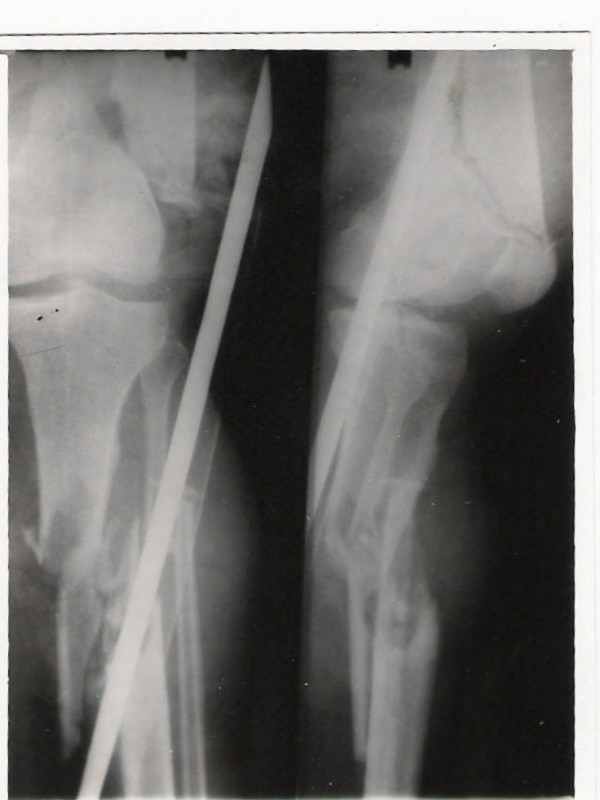
Type 2A Floating knee showing involvement of distal femur and proximal tibia.

**Figure 3:**
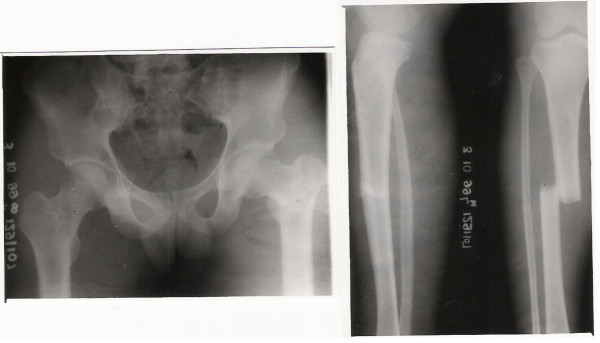
Type 2B Floating Knee showing involvement of the hip joint.

**Table 1:** Summary of patient profiles, treatment methods & final outcomes

**Patient No:**	**Age**	**Sex**	**Side**	**Type**	**Treatment**	**Complications**	**Final Outcome (Karlstrom criteria)**
1	40	M	R	2B	Other	None	Excellent
2	19	M	L	2B	Other	Infection	Poor
3	56	M	R	2B	Other	None	Excellent
4	27	M	R	2A	Other	Delayed union	Poor
5	24	M	R	1	Both IM nail	None	Excellent
6	42	M	L	2A	Other	None	Good
7	27	M	L	1	Both IM nail	Foot drop	Acceptable
8	24	M	R	1	Both IM nail	None	Excellent
9	21	M	R	1	Both IM nail	None	Excellent
10	32	F	L	2B	Other	Knee stiff	Good
11	30	M	R	2B	Both IM nail	None	Excellent
12	21	M	L	1	Other	Knee stiff	Good
13	35	M	R	1	Both IM nail	None	Excellent
14	22	F	R	1	Both IM nail	None	Good
15	20	M	L	1	Both IM nail	Delayed union	Poor
16	22	M	R	1	Both IM nail	Infection	Good
17	35	M	R	1	Both IM nail	None	Excellent
18	19	M	R	1	Both IM nail	None	Excellent
19	22	M	R	0	Both IM nail	None	Excellent
20	40	M	L	1	Both IM nail	None	Good
21	22	M	R	1	Both IM nail	None	Excellent
22	35	M	L	2A	Other	Knee stiff	Acceptable
23	24	M	R	1	Both IM nail	None	Good
24	25	M	R	1	Both IM nail	None	Excellent
25	18	M	R	2B	Both IM nail	None	Excellent
26	28	M	L	1	Other	Knee stiff	Good
27	22	M	L	1	Both IM nail	None	Excellent
28	31	M	R	1	Both IM nail	None	Excellent
29	26	M	R	1	Both IM nail	None	Good

**Table 2:** Blake and McBryde classification for Floating Knee injuries

Type 1 – True Floating Knee	The knee joint is isolated completely and not involved, with either shaft fractured.
Type 2 – Variant Floating knee	Involves one or more joints with either shaft fractured.
Type 2A	The knee joint alone is involved
Type 2B	Involves the hip or ankle joints

Patients were observed closely to detect development of fat embolism. (Tachypnoea, confusion, tachycardia). If fat embolism was diagnosed, patients were managed in the surgical intensive care and surgical fixation of the fractures was postponed. Patients with associated chest or head injuries were managed appropriately prior to surgical stabilisation of the fractures. We inserted chest drains in patients with suspected haemothorax or pneumothorax. All patients with fluctuating conscious levels had a CT scan of the brain. If an intracranial haematoma or bleed was diagnosed, these patients were referred to the neurosurgery unit for further management. Surgical stabilisation of the fractures was delayed till the head injury is dealt with. Detection of abdominal injuries was by clinical assessment and ultrasonography. If there was a suspicion of intra-abdominal injury, an urgent CT scan was indicated. If significant abdominal injuries were detected, these took priority over surgical stabilisation of the fractures. In our study, we did not have any patients with significant abdominal trauma, intracranial haematoma or bleed.

Surgical management of both the fractures were done once patients were hemodynamically stable and fit to undergo surgery. The femur fracture was fixed prior to the tibia fracture. Intramedullary nailing of both fractures was the commonest method. Both the femur and tibia nails were inserted antegrade. When external fixation was used in open tibia fractures, this was the definitive management. Associated injuries that needed surgery were treated under the same anaesthesia. Knee ligament injuries were diagnosed by clinical assessment by the surgeon after surgical stabilisation of the fractures. Lachman's test and posterior drawer's test were used to clinically assess the anterior and posterior cruciate ligaments respectively. If a knee ligament injury was suspected, a diagnostic arthroscopy was performed under the same anaesthesia and primary ligament reconstruction done. Patellar bone-tendon-bone grafts were used for reconstruction of the torn cruciate ligaments.

**Figure 4:**
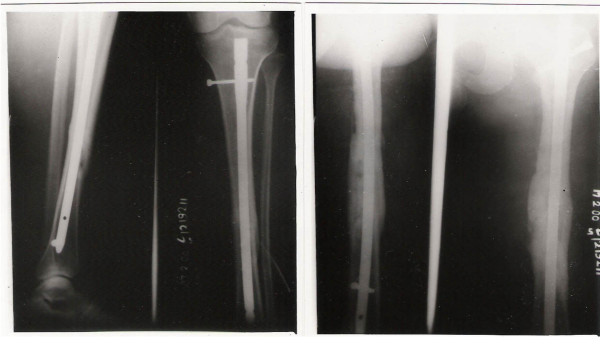
Fractures treated by intramedullary nailing showing union.

**Figure 5:**
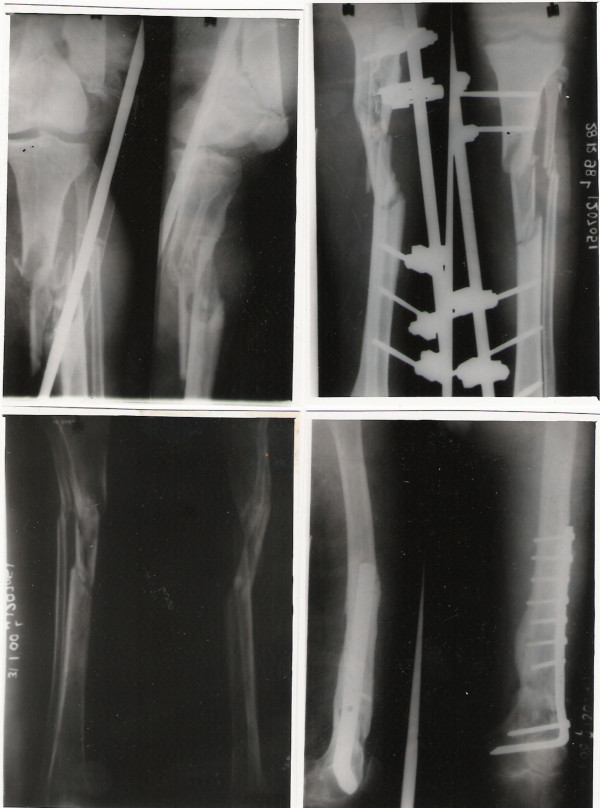
Complex Floating Knee injury treated with a Dynamic Condylar screw for the distal femur and external fixation for open comminuted tibia fracture at bony union.

Thromboprophylaxis was initiated in all patients in the post-operative period. Physiotherapy and mobilisation was started as soon as possible after surgery. Patients were followed up regularly till bony union (clinical and radiological). Functional assessment and final outcome was measured using the Karlstrom's criteria [11] after bony union [Table 3] [Figure 4 &5].

**Table 3:** Karlstrom criteria for functional assessment after management of floating knee injuries

CRITERION	EXCELLENT	GOOD	ACCEPTABLE	POOR
Subjective symptoms from thigh or leg	none	Intermittent slight symptoms	More severe symptom impairing function	Considerable functional impairment: pain at rest
Subjective symptoms from knee or ankle joint	none	Same as above	Same as above	Same as above
Walking ability	Unimpaired	Same as above	Walking distance restricted	Uses cane, crutch or other support
Work and sports	Same as before the accident	Given up some sport; work same as before accident	Change to less strenuous work	Permanent disability
Angulation, rotational deformity or both	0	<10 degrees	10 – 20 degrees	> 20 degrees
Shortening	0	< 1 centimetre	1 – 3 centimetres	> 3 centimetres
Restricted joint mobility	0	<10 degrees at ankle; <20 degrees at hip, knee or both	10 – 20 degrees at ankle; 20 – 40 at hip, knee or both	>20 degrees at ankle; >40 degrees at hip, knee or both

## Results

The mean age of the study group was 28 years (18 – 56).27 patients were involved in motor vehicle accidents while 2 patients sustained the injury by fall from height. The right side was involved in 19 and left side in 10 patients. There were 20 Type 1, 3 Type 2A and 6 Type 2B floating knee injuries (Blake & McBryde classification) [Table 2] [Figure 1, 2 &3]. There were 6 open fractures of the tibia – 4 – Type 3B and 2 – Type 2. The average time from admission to surgery was 2 days (Range 1–11). Surgery was delayed in 6 patients due to head injury and fat embolism. Intramedullary nailing for both fractures was performed in 20 patients. Combination of Dynamic hip screw, dynamic condylar screw, external fixation for tibia fracture and buttress plating for tibia plateau fractures were performed in the remaining 9 patients. The bony union times for the femoral and tibia fractures with different fixation methods were as detailed in Table 4.

**Table 4:** Bony union times for the femoral and tibial fractures with different fixation methods

Fracture fixation methods	Number of patients	Bony union time
Intramedullary nailing – Diaphyseal femur	20	19.1 weeks
Dynamic Hip screw – Proximal femur	5	15 weeks
Dynamic condylar screw – Distal femur	4	22.5 weeks
Intramedullary nailing – Diaphyseal tibia	20	20.8 weeks
Buttress plating – Tibial plateau	3	17 weeks
External Fixation – Open tibial fracture	6	28 weeks

38 associated injuries were noted in the 29 patients. These ranged from head injury to metatarsal fractures [Table 5]. 7 patients had ipsilateral knee injuries (3 patellar fractures, 2 anterior cruciate ligament tears, 1 posterior cruciate ligament tear and a bucket handle medial meniscal tear). 2 patients had haemopneumothoraces that needed tube thoracostomies. These patients underwent surgical stabilisation of the fractures without any delay. The chest drains were kept till the haemothorax was drained as monitored by serial chest radiographs. There was no delay in recovery or rehabilitation in these patients.

**Table 5:** Associated injuries with Floating knee and their management

Associated injury	Patients	Intervention
Patellar fractures	3	Open reduction internal fixation
Knee ligament injuries – Anterior cruciate, Posterior cruciate, Medial meniscus	4	Ligament reconstruction, medial meniscectomy
Clavicle fractures	4	Conservative
Femoral fractures (opposite)	3	Intramedullary nailing
Femoral artery block	1	Femoro-popliteal bypass graft
Humeral shaft fractures	4	Open reduction internal fixation
Head injury	3	Conservative
Rib fractures	1	Conservative
Haemo-pneumothorax	2	Chest drain insertion
Forearm bones fractures	1	Open reduction internal fixation
Contralateral tibial fractures	4	Intramedullary nailing
Tarsal/metatarsal fractures	4	Conservative
Fat embolism	3	Mechanical ventilation
Median/ulnar nerve palsy	1	Nerve conduction study

3 patients had contralateral femoral fractures and 4 had contralateral tibial fractures. 4 patients had associated humeral fractures and 1 patient had fractures of the forearm bones. 4 patients had tarsal/metatarsal fractures. All bony injuries that needed surgical stabilisation were managed during the same anaesthesia as for the surgical stabilisation of the floating knee.

3 patients sustained head injuries for which a CT scan of the brain was done. None of these patients had intracranial bleeds or haematomas that needed intervention by the neurosurgeons. All of these patients were diagnosed to have cerebral concussion. The initial assessment and management was neurological monitoring recording the pupil size and the Glasgow Coma Scale (GCS). These patients were operated only after their GCS was 15 and this delayed the time of initial surgery in all these patients by 2 – 3 days.

1 patient had a femoral artery injury which was suspected clinically and evaluated by a femoral angiogram. This revealed an intimal injury of the superficial femoral artery that needed a femoro-popliteal bypass graft which was performed by the vascular surgeons after surgical stabilisation of the fractures. Surgical stabilisation of the fractures was done initially to avoid placing stress on the vascular bypass graft during reduction of the fractures. The average surgical time was 1 hour more than in patients who needed surgical stabilisation of the floating knee alone. In some of these patients there was a delay in rehabilitation of 3 weeks on an average. 3 patients developed fat embolism and needed ventilatory support with monitoring in the Intensive care unit. The delay in surgery in these patients was 10 days (Range 8–11 days). The implication of the associated injuries is detailed in Table 6.

**Table 6:** Implications of associated injuries in the floating knee

Patient	Associated injury	Delay in primary surgery	Surgical duration (Hours)	Delay in rehabilitation
1	None	0	2:20	Nil
2	Cerebral concussion	2 days	2:00	Nil
3	Clavicle, Fat embolism	8 days	1:50	Nil
4	Patella	0	3:00	4 weeks
5	Contralateral femur	0	3:20	2 weeks
6	Anterior Cruciate	0	3:30	4 weeks
7	Clavicle, Humerus Forearm bones, Metatarsal	0	3:30	4 weeks
8	Medial meniscus	0	3:00	Nil
9	None	0	2:00	Nil
10	Contralateral tibia	0	2:30	2 weeks
11	Humerus	0	2:50	4 weeks
12	Fat embolism	11 days	1:50	Nil
13	Clavicle, Haemo-pneumothorax	0	2:10	1 week
14	Metatarsal	0	2:00	Nil
15	Humerus Radial nerve	0	3:00	4 weeks
16	Contralateral tibia, Fat embolism	9 days	2:40	2 weeks
17	Contralateral femur	0	2:50	2 weeks
18	Posterior Cruciate	0	3:20	4 weeks
19	Clavicle, Rib Haemo-pneumothorax	0	2:00	Nil
20	Patella, Metatarsal	0	2:40	4 weeks
21	None	0	1:50	Nil
22	Contralateral tibia Cerebral concussion	3	2:50	2 weeks
23	Humerus	0	3:00	3 weeks
24	Femoral artery injury	0	4:20	3 weeks
25	Contralateral tibia	0	3:00	2 weeks
26	Anterior Cruciate	0	3:20	4 weeks
27	Metatarsal	0	2:10	Nil
28	Contralateral femur Cerebral concussion	1	3:00	2 weeks
29	Patella	0	2:40	4 weeks

The complications encountered were knee stiffness in 4 patients, foot drop in 1 patient, delayed union of tibia in 2 patients and superficial infection in 2 patients. Nerve conduction study was done in the patient with foot drop which revealed an axonotmesis of the common peroneal nerve. The additional procedures were manipulation under anaesthesia for knee stiffness, dynamisation in one patient with delayed union and common peroneal nerve exploration in the patient with foot drop which revealed a partial nerve transection. Patients with delayed union needed dynamisation of the tibial nail and removal of external fixator and functional cast bracing of the fracture. These fractures went on to unite following these interventions. The superficial infections were related to pin sites of the external fixators which were managed by pin site care and antibiotics. The infection settled with this management. The average follow up was for 23.5 months (Range 21 – 26 months). In the assessment of end results after bony union according to the Karlstrom criteria the following results were obtained: Excellent – 15, Good – 9, Acceptable – 2 and Poor – 3.

## Discussion

When the knee joint is isolated partially or completely due to fracture of the femur and tibia the term "Floating Knee" is used [10]. Survivors of high-speed traffic accidents often have injuries to several of the parenchymal organs as well as multiple fractures. Careful evaluation of these injuries and resuscitation of the patient must precede the definitive management of specific fractures.

Hayes JT [5] suggested that automobile passengers with floating knee, braced their feet firmly against the sloping floor of the front seat just prior to the collision, their legs getting crumpled under the massive decelerating forces produced by the impact. Pedestrians were frequently catapulted some distance from the point of impact and were further injured by striking the pavement. In a study of 222 cases of floating knee by Fraser [2], all cases were involved in road traffic accidents.

Studies showed associated injuries like head injuries, chest injuries, abdominal injuries and injuries to other extremities. Most of the injuries to the head, chest and abdomen were life threatening. Adamson et al in their study encountered 71% major associated injuries with 21% vascular injuries [12]. The reported mortality rate ranged from 5% – 15%, reflecting the seriousness of the associated injuries [1]. Deliberate and careful examination of the patient must be carried out in order to determine whether a major intracranial, abdominal or thoracic injury is present. Such injuries should take precedence over extremity injuries in the priority of treatment.

There are plenty of studies in the literature detailing different management options for the Floating Knee. Hayes JT [5] opined that in a patient with multiple fractures in the same extremity, operative fixation of one or more of the fractures was valuable in the management of the entire limb. Ratcliff AH [8] found that internal fixation of both the fractures should be done wherever possible as these patients were less likely to develop knee stiffness or shortening and were in hospital and off work for less time than those treated conservatively. Omer GE [6] treated the Floating Knee by both conservative and operative fixation found that where internal fixation was done for both femoral and tibial fractures, the healing time was about 8 weeks earlier than the group managed conservatively. Behr JT [3] treated patients with the Floating knee by closed intramedullary nailing with Ender nails and achieved femoral union at an average of 10.3 weeks and tibial union at 18 weeks. Ostrum RF [7] treated patients with a retrograde femoral tibial intramedullary nail through a 4 cm medial parapatellar incision. The average time to union of the femoral fractures was 14.7 weeks and that for the tibial fractures was 23 weeks. They opined that this method was an excellent treatment option.

The general consensus in recent studies is that the best management for the Floating knee is surgical fixation of both the fractures with intramedullary nails. Dwyer used combined modalities of treatment with one fracture managed conservatively and the other surgically. They concluded that the treatment method for the tibia did not interfere with joint mobilisation [13]. Lundy recommended surgical stabilisation of the fractures for early mobilisation which produced the best results [14]. Theodoratus recommended intramedullary nailing as the best choice of treatment except for grade 3B & C open fractures [15]. Single incision technique for nailing of both the fractures have been recommended by several authors [7,16,17]. Rios J compared single incision versus traditional antegrade nailing of the fractures and found the former to have less surgical & anaesthesia time with reduced blood loss [17]. Shiedts found an increased incidence of fat embolism when both fractures were treated by reamed nails [18].

Szalay [19] demonstrated knee ligament laxity in 53% of patients while 18% complained of instability. Most of the patients with instability had a rupture of the anterior cruciate ligament with or without damage to other ligaments. They concluded that knee ligament injury was more common with floating knee injuries than with isolated femoral fractures and advocated careful assessment of the knee in all cases of fractures of the femur and floating knee injuries. Other studies [20] have showed that the incidence of knee ligament injuries in the floating knee was upto 50%, most of which were missed in the initial assessment. Meticulous examination of the knee at the time of injury is strongly advocated although the practicality of this method is questionable.

Our study showed a male predominance comparable to other studies. Most of the studies showed road traffic accidents as the only mode of injury. In our study, the most common mode of injury was road traffic accidents but two of our patients sustained their injury after a fall from height. This mode of injury for the Floating Knee has not been mentioned in the literature reviewed. The classification used by us was the one that was proposed by Robert Blake [10]. This was used as it took into account the injuries sustained at the hip or ankle of the affected side and helps one in planning the surgical procedure. The other classification system advocated by Fraser [2] includes intra-articular fractures at the knee but does not mention about injuries to the ipsilateral hip or ankle both of which can have implications on the surgical management of the Floating Knee. Our management consisted of treating both the femoral and tibial fractures surgically, most of them by intramedullary nailing using an interlocking nail. With this management, we found the fracture union time and functional recovery was better than the other surgical modalities. This was in accordance to studies by Gregory [4] and Ostrum RF [7] who had excellent results with fixation of both fractures by intra-medullary nailing. Both these authors used a retrograde nailing for the femur although in our study all the nailing was antegrade. Though no knee problems have been found when single incision technique is used [4,7,16] we feel that antegrade nailing allowed easier knee ligament reconstruction if needed as the femoral nail inserted retrograde would make knee ligament reconstruction technically difficult.

Intra-articular involvement of the fractures, higher skeletal injury scores and severity of soft tissue injuries are significant indicators of poor outcome results [21-23]. Hee suggested a preoperative scoring system which took into consideration the age, smoking status at time of injury, Injury severity scores, open fractures, segmental fractures and comminution to prognosticate the final outcome of these fractures [24].

The best results were seen when both fractures were treated by intramedullary nailing. We found that these patients returned to their normal level of activity earlier than when the fractures were treated with other modalities. Tibia fractures treated with external fixation had a longer union time probably related to the soft tissue injury and comminution at the initial injury. The 3 patients who had a poor outcome in our study were 2 patients with tibia plateau fractures who had knee stiffness and persisting pain in the knee while the other patient had a Grade 3B open tibia fracture treated by external fixation. This shows that the poor prognostic factors were related to the type of fracture (open or closed, intra-articular fractures, severe comminution). The associated injuries played a major role in the initial outcome of patients in our study with regards to delay in initial surgery, prolonged duration of surgery, anaesthetic exposure and delay in rehabilitation. From our study we found Floating knee injuries to be a group of complex injuries that needed careful assessment to detect poor prognostic factors (open, intra-articular, comminuted fractures) and associated injuries, surgical fixation of the fractures with thorough planning of surgeries and prolonged rehabilitation. Combination of all these would determine the ultimate outcome of these patients.

## Conclusion

The Floating Knee is a complex injury with more than just ipsilateral fractures of the femur and tibia. The associated injuries and the type of fracture (open, intra-articular, comminution) are prognostic indicators of the initial and final outcome in patients. We recommend thorough initial assessment of patients with regards to life threatening associated injuries, surgical fixation of both fractures preferably by intramedullary nailing, knee ligament assessment to detect injuries and rigorous post-operative rehabilitation for a good final outcome.

## Competing interests

The author(s) declare that they have no competing interests.

## Authors' contributions

UR was involved in conducting the study, collecting patient details, reviewing the literature, drafted the manuscript and proof read the manuscript. RSY was involved in reviewing the literature and proof read the manuscript. RN is the senior author and was responsible for final proof reading of the article. All authors have read and approved the final manuscript.
